# Scaling up a virginiamycin production by a high-yield *Streptomyces virginiae* VKM Ac-2738D strain using adsorbing resin addition and fed-batch fermentation under controlled conditions

**DOI:** 10.1007/s13205-016-0566-8

**Published:** 2016-11-12

**Authors:** Vakhtang Dzhavakhiya, Vyacheslav Savushkin, Alexander Ovchinnikov, Vladislav Glagolev, Veronika Savelyeva, Evgeniya Popova, Nikita Novak, Elena Glagoleva

**Affiliations:** INGBIO Innovative Enterprise, Pr. 60-letiya Oktyabrya, 7/1, Moscow, 117312 Russia

**Keywords:** *Streptomyces virginiae*, Virginiamycin, Fed-batch fermentation, Scale-up, Dissolved oxygen, Synthetic resins

## Abstract

Virginiamycin produced by *Streptomyces virginiae* as a natural mix of macrocyclic peptidolactones M and S is widely used in the industrial production of ethanol fuel and as an antibiotic feed additive for cattle and poultry. Its main antimicrobial components, M1 and S1 factors, act synergistically if the M1:S1 ratio in the final product is 70–75:25–30. This fact significantly complicates the development of stable high-yield strains suitable for industrial application. In the previous work, authors obtained a mutant *S. virginiae* VKM Ac-2738D strain, characterized by a high productivity in flasks and the optimum M1:S1 ratio (75:25) in the final product. In this study, the scale-up of the virginiamycin production by VKM AC-2738D from shake flasks to a pilot-scale (100 L) stirred fermentor was carried out and the possibility of the in situ use of synthetic adsorbing resins to remove virginiamycin from culture broth was assessed. After the optimization of pH and dissolved oxygen concentration (6.8–7.0 and 50%, respectively), the fed-batch fermentation of VKM Ac-2738D with continuous addition of 50% sucrose solution (5 g/L/day starting from 48 h of fermentation) resulted in a final virginiamycin titer of 4.9 g/L. Among four tested resins, Diaion^®^ HP21 added to fermentation medium prior to sterilization absorbed 98.5% of the total virginiamycin that simplifies its further recovery procedure and increased its total titer to 5.6 g/L at the M1:S1 ratio of 74:26. The developed technology has several important advantages, which include (1) the optimum M1:S1 ratio in the final product, (2) the possibility to use sucrose as a carbon source instead of traditionally used and more expensive glucose or d-maltose, and (3) selective binding of up to 98.5% of produced virginiamycin on the adsorbing resin.

## Introduction

Virginiamycin, an antibiotic produced by *Streptomyces virginiae*, belongs to the streptogramin group and represents a natural mix of two synergistic factors, M (hybrid polyketide-peptide) and S (peptide). The main antimicrobial components of the antibiotic are the M1 and S1 factors. One of the unique features of the virginiamycin biosynthesis is a simultaneous production of both M1 and S1 at a ratio providing their maximum synergistic activity in the suppression of the protein biosynthesis in susceptible microorganisms (Di Giambattista et al. 1989). The maximum synergism is observed when M1 and S1 are present in the optimum ratio of 70–75:25–30; in this case, the total antimicrobial activity of virginiamycin increases 3.5–4 times (Mast and Wohlleben 2014). There is also another point of view based on the study of antimicrobial characteristics of quinupristin/dalfopristin, another antibiotic of the virginiamycin group, at different ratios of its components (Jamjian et al. 1997). This study showed no significant difference between various quinupristin/dalfopristin ratios. Nevertheless, there were no similar studies concerning virginiamycin, i.e., the idea of the best synergistic ratio of its components still has not been disproved.

Virginiamycin M1 and S1 alone exhibit bacteriostatic activity, but their synergic combination possesses bactericidal activity (Mast and Wohlleben 2014). In addition, virginiamycin can be used as a growth stimulator, since it optimizes the absorption and metabolism of nutrients, improves the state of small intestine epithelium, and inhibits the synthesis of harmful toxins and metabolites by gut organisms (Feighner and Dashkevicz 1987; Cervantes et al. 2002).

Virginiamycin is actively used for the industrial production of ethanol fuel (Hynes et al. 1997; Arshad et al. 2011); in addition, some countries, including USA, Russia, Brazil, and China, allow its use as an antibiotic feed additive for cattle and poultry (Hofacre et al. 1998; Singh et al. 2008; Shojadoost et al. 2013). By 2020, the predicted increase in the consumption of this antibiotic in North America, Europe, and Asia will be 6% (Chem-Report 2015).

To produce a sufficient volume of virginiamycin, the development of high-yield industrial *S. virginiae* strains and improved technologies for their cultivation are required. However, the biosynthesis of virginiamycin components is controlled by a lot of different regulators (Pulsawat et al. 2009), and its antibiotic efficiency depends on the correct M1:S1 ratio, so the development of stable overproducing strains seems to be an extremely difficult task. An indirect evidence of this fact is a very small number of publications on the strain improvement of *S. virginiae*. The productivity of strains reported in various publications is relatively low and varies from 0.28 to 1.9 g/L (Biot 1984; Prikrylova et al. 1987; Zvenigorodskii et al. 2001; Zhao et al. 2011). An additional increase of the strain productivity can be achieved by medium improvement and optimization of fermentation conditions, but even in this case the productivity of the existing industrial strains remains mainly within the range 3–4 g/L (Biot 1984; Zhao et al. 2011; Han et al. 2013). Thus, the problem of the development of overproducing *S. virginiae* strains and technologies for the industrial biosynthesis of virginiamycin is still relevant.

In our previous study, a high-yield *S. virginiae* strain, characterized by the basic productivity of about 2.6 g/L and the optimal M1:S1 ratio in the final product, has been obtained by induced UV mutagenesis from the VKPM Ac-790 strain, whose productivity was only 0.6 g/L (Dzhavakhiya et al., unpublished manuscript). After the completion of the strain identification and the study of its biochemical and physiological properties, the strain was deposited at the All-Russian Collection of Microorganisms (Skryabin Institute of Biochemistry and Physiology of Microorganisms, Pushchino, Russia) under the accession no. VKM Ac-2738D.

After the medium improvement, the productivity of new strain in flasks reached 4.3 g/L, while the M1:S1 ratio remained synergistic (75:25). One should note that the majority of publications and patents describing the development of virginiamycin-producing strains do not contain information on the determination of this ratio in the synthesized product. At the same time, possible violation of the synergistic M1:S1 ratio during the virginiamycin biosynthesis may result in the necessity of additional technological stages intended to either correct this ratio, or to separate M1 and S1 components during the isolation and purification process for their further mixing in the required proportion in the final formulation. Therefore, the VKM Ac-2738D strain has an additional advantage that increases its value for the industrial production of virginiamycin.

An increased productivity of industrial antibiotic-producing strains often results in a self-inhibiting effect of their metabolites able to suppress not only their own biosynthesis, but also the growth and development of a microorganism. One of the ways to reduce possible cytotoxicity and self-inhibition effects of accumulated target product is fermentation in the presence of adsorbing resins able to selectively remove the product from the culture broth (Phillips et al. 2013). However, this method requires the selection of a certain resin type for each metabolite, since even resins with similar characteristics can provide opposite results, either increasing, or reducing the content of a target product in culture broth (Lam et al. 1995; Warr et al. 1996). A correct adsorbent selection can provide a very significant (sometimes 100-fold) yield increase (Singh et al. 2010). In spite of a large number of publications describing the use of adsorbing resins in the microbial production of biologically active substances, we did not find any information on the use of such resins in the virginiamycin production, though the use of adsorbing resins was reported to increase the production of a similar antibiotic, pristinamycin, by 1.25–1.55 times (Jia et al. 2006, Zhang et al. 2012).

The purpose of this study was the scale-up of the virginiamycin production by *S. virginiae* VKM Ac-2738D from shake flasks to a pilot-scale (100 L) stirred fermentor and the assessment of the possibility of in situ use of synthetic adsorbing resins to bind produced virginiamycin.

## Materials and methods

### Virginiamycin-producing microorganism and media composition

The *S. virginiae* VKM Ac-2738D strain obtained from the known VKPM Ac-790 strain by a multi-step UV mutagenesis and deposited at the All-Russian Collection of Microorganisms was used as a virginiamycin producer. The productivity of the strain on the basic fermentation medium was 2.6 g/L.

The strain was maintained and stored on a modified Gauze’s agar medium No. 1 of the following composition (g/L): agar, 20; corn starch, 20; pea flour, 10; KH_2_PO_4_, 0.5; MgSO_4_, 0.5; NaCl, 0.5; FeSO_4_, 0.01; KNO_3_, 1.0 (pH 6.8–7.0). The same medium was used for the seed culture preparation.

Vegetation medium for seed cultures consisted of the following components (g/L): glucose, 1.0; soluble starch, 10.0; meat extract, 3.0; yeast autolysate, 1.0; casein hydrolyzate, 5.0, CaCO_3_, 0.5 (pH 6.8–7.0).

Basic fermentation medium consisted of the following components (g/L): sucrose, 50.0; pea flour, 10.0; corn gluten, 5.0; fermentative peptone, 2.5; yeast extract, 5.0; malt extract, 10.0; NaCl, 3.0, MgSO_4_, 0.5; CaCO_3_, 5.0 (pH 6.8–7.0). The medium was sterilized for 1 h at 121–123 °C by direct steam treatment in a fermenter.

### Seed culture preparation and fermentation conditions

Seed culture used for the fermenter inoculation passed two generations. Spore suspension was prepared from a 7- to 10-day agar culture grown at 28 °C, and inoculated into 50-mL Erlenmeyer flasks containing 10 mL of vegetation medium. The culture was grown for 24 h at 28 °C on Innova 44 incubation shakers (New Brunswick, USA) at 220 rpm (5-cm orbit) and then reinoculated into 2-L flasks containing 500 mL of vegetation medium. Culture growth was carried out under the same conditions as described above. The grown second-generation culture was inoculated into a fermenter. Fermentation was carried out in a 100-L stirred fermenter (Profsplav, Russia) for 96 h at 28 °C under controlled ranges of pH (4–9) and dissolved oxygen (DO) concentration (10–70%). The required DO concentration was adjusted by automatically changing the agitation speed and by regulating the air supply.

### Adsorbing resin assessment

Four types of synthetic resins were assessed for their possibility to bind virginiamycin: Diaion^®^ HP20, Diaion^®^ HP21 (Sorbent Technologies, USA), Amberlite^®^ IR120, and Amberlite^®^ IRA900 (Acros Organics, Belgium). The resins were added to fermentation medium prior to sterilization at a concentration of 20 g/L.

During preliminary assessment in flasks, 1 ml of seed material was inoculated into 50-mL Erlenmeyer flasks containing synthetic resin (20 g/L) and 10 mL of fermentation medium of the following composition (g/L): sucrose, 35.0; pea flour, 10.0; corn gluten, 5.0; meat peptone, 2.5; yeast extract, 1.0; malt extract, 10.0; CaCO_3_, 5.0; KH_2_PO_4_, 1.6; Na_2_HPO_4_, 1.0; (NH4)_2_SO_4_, 1.0; MgSO_4_, 1.0; NaCl, 2.0 g/L (pH 7.0–7.2). The culture was grown for 96 h at 28 °C on Innova 44 incubation shakers (New Brunswick, USA) at 250 rpm (5-cm orbit). At the end of fermentation, the content of free and adsorbed virginiamycin was determined by HPLC as described below.

### Biomass content assessment

The biomass content in culture broth was determined by centrifugation. Culture broth samples were weighed, added into weighed centrifuge tubes, and centrifuged at 4500 rpm for 10 min on a Sigma 2-16P centrifuge (Sigma, Germany). After the removal of a supernatant, each tube was weighed again. The biomass content in culture broth was calculated using the following formula:

$$ X = \frac{b}{a} \cdot 100\% , $$

where *b* is the mass of a precipitate after centrifugation and *a* is the mass of the tested culture broth sample.

### Determination of free and adsorbed virginiamycin content by HPLC

Virginiamycin was extracted from culture broth samples by adding an equal volume of ethyl acetate followed by a 2-h incubation under constant stirring. Then the ethyl acetate layer was separated and centrifuged at 12,000 rpm for 3 min. From each sample, 200 µL of supernatant was taken, dried, dissolved in 400 µL of the acetonitrile:water mix (55:45), and analyzed by HPLC.

To determine the level of virginiamycin absorption by tested resins, resin-containing culture broth was filtered through a stainless steel mesh (hole size <0.1 mm), and the collected resin was washed with distilled water. Further virginiamycin extraction and sample preparation were carried out separately for resin and culture broth as described above.

Virginiamycin content in prepared samples was determined by HPLC. The analysis was performed using an Agilent 1200 chromatographic system (Agilent Technologies, USA) with a Zorbax SB-C18 column (250 × 4.6 mm, Agilent Technologies, USA) filled with octadecyl silica gel (5 µm). The mobile phase was acetonitrile:water (55:45) acidified by addition of 100 μL of acetic acid, the flow rate was 1.0 mL/min at 40 °C. Standard preparations of virginiamycin M1 BTZ 2012379 and S1 BTZ 2012376 (Sigma-Aldrich, USA) dissolved in the mobile phase were used as reference samples. The sample volume was 10 µL. The absorbance was measured at 220 nm. The retention times for M1 and S1 were 6.93 and 11.92 min, respectively.

### Statistical analysis

Each experiment was arranged in 3–4 replications. The statistical treatment of obtained data was carried out using the MS Excel 2003 program.

## Results

### Cultivation of* S. virginiae* VKM Ac-2738D under registration of basic technological parameters

**Fig. 1:**
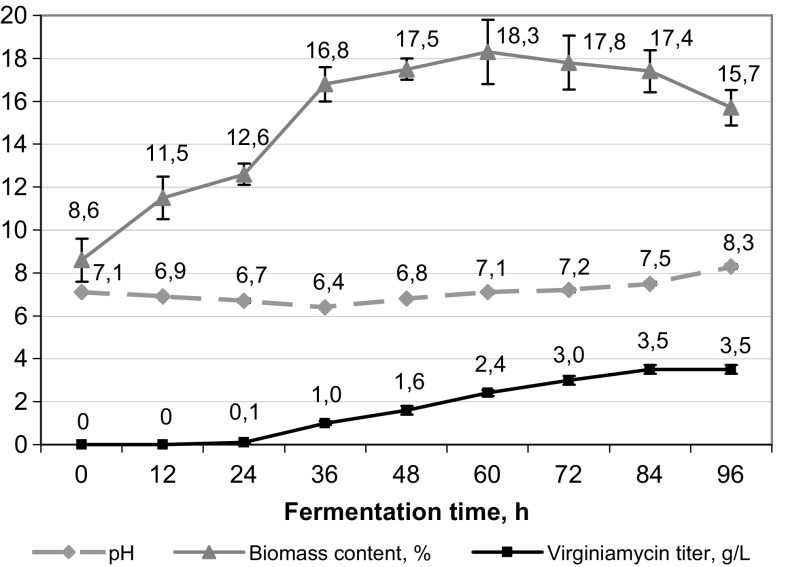
Changes in the medium pH, virginiamycin titer, and biomass accumulation in culture broth during the fermentation of *Streptomyces virginiae* VKM Ac-2738D under the mode of registration of basic technological parameters

In the first experiment, strain cultivation was carried out under standard conditions. Air supply was maintained at the level of 0.5 L/L/min, the speed rate of the impeller was maintained at 250 rpm. During the first 36 h of fermentation, pH decreased from 7.1 to 6.4, and then increased reaching 8.3 at the end of the process (Fig. 1). At the 60th hour of fermentation, crude biomass content in culture broth increased from 8.6 to 18.3% and then decreased to 15.7% that probably indicated cell lysis. This hypothesis was also confirmed by a pH increase up to 8.3 observed at 92–96 h of fermentation: when carbon source is depleted and its concentration falls below the level required for the cell maintenance, it results in cell death and lysis followed by protein hydrolysis and corresponding liberation of ammonia that increases pH (Schaffner and Toledo 1991; Ting et al. 2008). At the end of fermentation (96 h), virginiamycin content in culture broth reached 3.5 ± 0.2 g/L. The further fermentation at pH 8.3 resulted in the degradation of virginiamycin and the corresponding decrease of its titer.

**Fig. 2:**
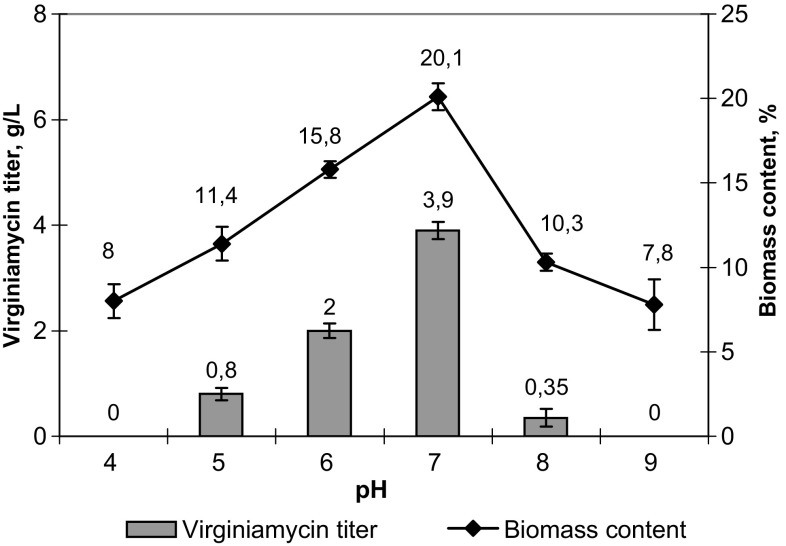
Effect of medium pH on the biomass accumulation and virginiamycin titer in culture broth of *Streptomyces virginiae* VKM Ac-2738D after 84 h of fermentation

Weak biomass accumulation indicated insufficient content of carbohydrates in fermentation medium and probable negative effect of increased pH on the virginiamycin biosynthesis. To check the hypothesis on the negative effect of increased pH on the biosynthesis of the target product and to determine the optimum pH value, a series of fermentations was carried out at different pH values within the range of 4–9. The tested pH values were maintained by an automatic HCl supply via a peristaltic pump. Both control parameters were registered after 84 h of fermentation. The maximum virginiamycin titer (3.90 ± 0.16 g/L) and biomass content in culture broth (20.1 ± 0.8%) were observed for pH 7.0 (Fig. 2). In all further experiments, the medium pH was maintained at the level of 6.8–7.0. According to our observations, pH stabilization allowed the further prolongation of fermentation by 24 h more, but did not result in an increased virginiamycin titer, so the fermentation time exceeding 96 h was rather unprofitable.

### Productivity of* Streptomyces virginiae* VKM Ac-2738D under controlled pH and dissolved oxygen concentration

**Fig. 3:**
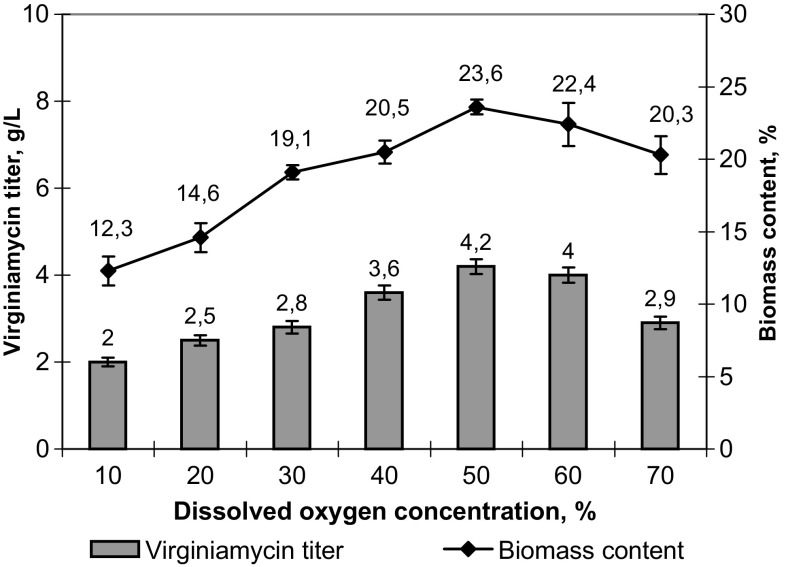
Effect of a dissolved oxygen concentration on the biomass accumulation and virginiamycin titer in culture broth of *Streptomyces virginiae* VKM Ac-2738D after 84 h of fermentation under controlled pH conditions (pH 6.8–7.0)

The DO concentration in fermentation medium is one of the most important factors influencing the biosynthesis of virginiamycin (Shioya et al. 1999). Therefore, an experiment was arranged to evaluate the effect of different DO concentrations on the biomass accumulation and productivity of VKM Ac-2738D under controlled pH (6.8–7.0). The DO level was adjusted by manipulating the rotation speed of impeller and changing of the air supply. According to the obtained results, the maximum productivity (4.20 ± 0.17 g/L) and biomass accumulation (23.6 ± 0.5%) were observed at the DO concentration equal to 50% (Fig. 3). This optimal DO value was used for further experiments.

### Productivity of *Streptomyces virginiae* VKM Ac-2738D under fed-batch fermentation conditions with controlled addition of sucrose

Metabolic changes in overproducing mutant strains often affect the possibility to utilize various nutrients. In addition, high-yield strains are sometimes characterized by more intensive growth, so they may need higher concentrations of nutrients, especially carbon sources (Zhdanova 1989). Therefore, it is important to select the most efficient types and concentrations of these nutrients for each industrial strain to realize its productivity potential.

In the case of *S. virginiae*, the most available carbon sources are simple sugars, such as mono- and disaccharides. For example, it was shown that the optimal additional carbon source for *S. virginiae* ATCC 13161 was d-maltose, which doubled virginiamycin production (Zvenigorodskii et al. 2001). In the same study, good results were also obtained for mannose and d-glucose (yield increase by 50% of the control), but the use of complex carbohydrates, such as starch resulted in a lower effect. Fructose and glucose were shown to be the best carbon sources for the *S. virginiae* HM3 strain (Rifaat and Kansoh 2005) and six other *S. virginiae* strains (Anzai et al. 1994); in the last case, four strains were also able to utilize L-arabinose. Boeck et al. (1985) described the *S. virginiae* NRRL 15156 strain able to grow on cellobiose, fructose, galactose, glucose, maltose, ribose, salicin, and succinate.

One of distinctive features of VKM Ac-2738D was its ability to utilize sucrose as the carbon source, whereas the parental strain and all above-mentioned strains were not able to grow on sucrose. Comparing to other carbon sources tested, sucrose provided the maximum virginiamycin titer (3.27 g/L) and M1:S1 ratio of 72:28; a high productivity level was also observed for glucose and starch (3.15 and 2.95 g/L, respectively), but in both cases, the M1:S1 ratio was shifted to 55:45 and 59:41, respectively (Dzhavakhiya et al. unpublished manuscript). Therefore, sucrose was selected as the additional carbon source.

Rapidly metabolized simple sugars, including sucrose, are able to interfere with the biosynthesis of many antibiotics resulting in so-called carbon catabolite repression (Sanchez and Demain 2002). The possible solution of this problem is a fed-batch fermentation, since the concentration of the limiting substrate may be maintained at a very low level avoiding repressive effects of high substrate concentration and improving the final antibiotic yield (Callewaert and de Vuyst 2000; Elsayed et al. 2015). Taking into account this fact, a continuous addition of 50% sterile sucrose solution was provided after 48 h of fermentation under controlled optimal pH and DO concentration (6.8–7.0 and 50%, respectively). The sucrose addition started in automatic mode when the pH value exceeded 6.8.

**Fig. 4:**
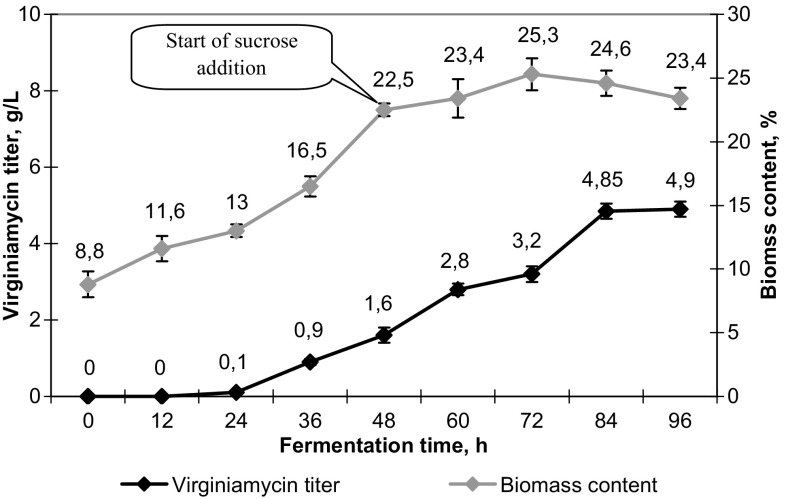
Effect of fed-batch fermentation of *Streptomyces virginiae* VKM Ac-2738D with addition of 50% sucrose solution on the biomass content and virginiamycin titer in culture broth under controlled pH (6.8–7.0) and dissolved oxygen concentration (50%)

A continuous supply of 50% sucrose solution during the fermentation process provided additional pH stabilization due to the formation of organic acids during metabolic processes. This pH stabilization resulted in the prolongation of the active virginiamycin biosynthesis providing the final virginiamycin titer reaching 4.9 ± 0.2 g/L (Fig. 4). The biomass content in culture broth reached maximum after 72 h of fermentation (25.3 ± 1.3%) and then slightly decreased to the end of fermentation (23.4 ± 0.8%). The average sucrose consumption by the culture made 5 g/L/day.

### Evaluation of synthetic adsorbing resins

Four synthetic resins of different types were evaluated for their ability to bind virginiamycin in situ and to improve the productivity of *S. virginiae* VKM Ac-2738D; the M1:S1 ratio in the final free and bound product was also assessed. The results of a preliminary comparative evaluation of resins in 50-mL flasks are shown in Table 1.

**Table 1:** Effect of different types of adsorbing resins on the virginiamycin M1 and S1 biosynthesis by *S. virginiae* IB 25-8 and the level of its adsorption from culture broth

Resin type	Adsorbed virginiamycin			Free virginiamycin			Strain productivity, g/L
	% of the total amount	M1, %	S1, %	% of the total amount	M1, %	S1, %	
Control	–	–	–	100	78	22	3.97
Diaion^®^ HP20	65	63	37	35	63	37	4.25
Diaion^®^ HP21	98.5	74	26	1.5	70	30	5.05
Amberlite IRA900	12	89	11	88	90	10	2.25
Amberlite IR120	3	25	75	97	20	80	2.95

According to the obtained results, Diaion^®^ HP21 provided the best result in relation to both total strain productivity and M1:S1 ratio in bound virginiamycin (5.05 g/L and 74:26, respectively). Note that this resin demonstrated the highest virginiamycin adsorption (98.5% of the total yield) able to significantly facilitate the further antibiotic recovery. The M1:S1 ratio in free virginiamycin (1.5%) also remained within the desired interval (70:30).

**Fig. 5:**
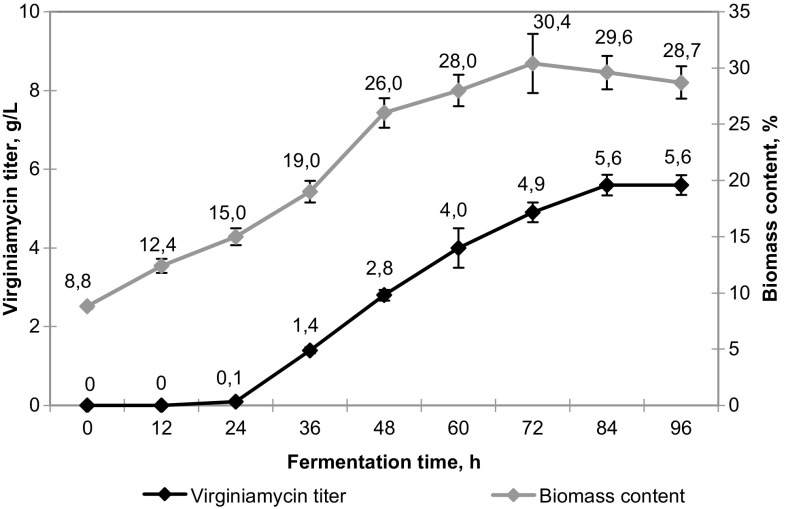
Biomass accumulation and virginiamycin production by *Streptomyces virginiae* VKM Ac-2738D under fed-batch fermentation in the presence of a Diaion^®^ HP21 adsorbing resin (20 g/L). Fermentation conditions included controlled pH (6.8–7.0) and dissolved oxygen concentration (50%) and the continuous addition of 50% sucrose solution after 48 h of culture growth (5 g/L/day)

At the next step, the effect of in situ addition of the selected sorbent on the productivity of VKM Ac-2738D was evaluated under fed-batch fermentation. Fermentation conditions corresponded to those optimized in the previous experiments (controlled pH and DO concentration and continuous feeding with 50% sucrose solution at a rate of 5 g/L/day starting from the 48th h). The addition of HP21 to fermentation medium increased productivity to 5.6 ± 0.3 g/L and biomass accumulation to 29–30% (Fig. 5) that may be due to virginiamycin adsorption and, therefore, less self-inhibitory effect.

## Discussion

As we have mentioned earlier, a complex nature of virginiamycin significantly complicates the development of overproducing strains. The analysis of virginiamycin-related publications and patents showed that the problem of development of high-yield industrial strains able to synthesize both virginiamycin M1 and S1 in the desired synergistic ratio is still relevant, since the M1:S1 ratio in the final product can significantly change during strain improvement that can negatively influence on the antimicrobial activity of a target product. For example, in the course of a long-term program of extensive selection of *S. virginiae* performed by the Phibro Animal Health corporation (the main world manufacturer of virginiamycin-based formulations), the use of UV and chemical mutagenesis resulted in a wide range of mutant strains with different characteristics, including various deviations in the M1:S1 ratio or even completely blocked biosynthesis of one or both components (Lanoot et al. 2005). Since the vast majority of the mentioned publications do not contain information on the control of the M1:S1 ratio, the VKM Ac-2738D strain, which provides the optimum value of this parameter, seems to be especially valuable for the industrial production of virginiamycin.

As a rule, microbial biosynthesis technologies are first developed under laboratory conditions and then scaled-up in a pilot and then industrial scale. The main task of the scale-up process is to increase the volume of production while maintaining or even increasing the productivity of the used strain (Schmidt 2005). A successful scaling-up strongly depends on fermentation conditions. High cell density in fermenters significantly differs from their natural growth conditions and results in some stresses related to various environmental factors, such as changes in temperature, pH, osmotic concentrations, etc. (Mattanovich et al. 2004). Substrate and DO concentrations also influence on the cell growth and biosynthetic processes (Mattanovich et al. 2004; Garcia-Ochoa and Gomez 2009).

The number of existing publications and patents related to the technologies of the industrial virginiamycin biosynthesis is relatively low. The majority of such publications are devoted to the optimization of the medium components, including carbon/nitrogen sources and minor elements (Zvenigorodskii et al. 2001; Zhao et al. 2011; Yong 2015); some authors also describe the effect of addition of precursors (Han et al. 2013) and regulators of the virginiamycin biosynthesis (Yang et al. 1995). The optimization of fermentation conditions is described only in several studies. One group of authors demonstrated the importance of the DO concentration and agitation rate control for the increase of a virginiamycin production by a low-yield *S. virginiae* MAFF 10-06014 (Yang et al. 1996; Shioya et al. 1999). The optimization of these two parameters combined with the addition of the virginiae butanolide C (VB-C), which regulates biosynthesis of virginiamycin, provided an 18× increase in the virginiamycin M1 production. Another group of authors described in their patent an increased virginiamycin production under conditions of fed-batch fermentation with continuous or intermittent addition of 1–5% glucose and either mixture of amino acids, or their source (Nomura et al. 1990).

In this study, we assessed the effect of such fermentation parameters as the medium pH, DO concentration, and continuous addition of 50% sucrose solution on the virginiamycin productivity of VKM Ac-2738D. The pH maintenance at the optimal level (6.8–7.0) provided an antibiotic titer increase to 3.9 g/L (+11.4%), an additional DO control at a level of 50% improved it up to 4.2 g/L (+20%), and the fed-batch fermentation strategy using 50% sucrose solution resulted in the virginiamycin titer increase up to 4.9 g/L that was 40% higher than that under initial fermentation conditions (3.5 g/L).

In addition, we also evaluated the possibility of the use of synthetic adsorbing resins for selective binding of virginiamycin and reduction of a self-inhibition effect. In spite of a wide use of this approach in the microbial production of biologically active substances including pristinamycin, an antibiotic, which is very similar to virginiamycin (Jia et al. 2006; Zhang et al. 2012), authors did not find any data for virginiamycin. The only known patent, mentioning the use of an unknown Dow macroporous resin in the virginiamycin biosynthesis, described the application of this resin as a tool to improve gas exchange in the fermentation medium (Yong 2015). According to this patent, the addition of a Dow resin and a surfactant combined with the performed medium optimization provided the virginiamycin titer exceeding 6 g/L; however, this information does not allow us to evaluate either relative productivity increase caused by the resin addition, or the M1:S1 ratio in the final product. At the same time, our study of several adsorbing resins showed a variability of this ratio depending on the resin type and revealed a sorbent, which use provided an increase of the virginiamycin titer up to 5.6 g/L; the M1:S1 ratio in the bound virginiamycin remained within the optimum range (74:26). Moreover, the Diaion^®^ HP21 resin, added to fermentation medium prior the sterilization, provided a selective binding of almost all virginiamycin from culture broth facilitating its further recovery.

The revealed difference in the final M1:S1 ratio depending on the resin type represents an interesting phenomenon that has not been described for the production of either virginiamycin, or any other similar antibiotic. Now authors do not have clear explanation of this fact. It may be caused by some steric factors providing different levels of adsorption of M1 and S1 components by different resins. Alternatively, since resins are added to the medium before fermentation, they can adsorb some nutrients or metabolites important for the biosynthesis of virginiamycin M1 or S1 components; the corresponding deficiency may result in the shift in the ratio of synthesized components. An additional study of this phenomenon it required.

Thus, we successfully scaled-up the technology of a virginiamycin production by high-yield *S. virginiae* VKM Ac-2738D from 50-ml shake flasks to a 100-L stirred fermenter and reproduced on a pilot-scale results obtained on a lab-scale. Different types of synthetic resins were first evaluated for their ability to reduce the self-inhibition effect and improve the virginiamycin titer in a lab-scale, and an optimal variant was successfully tested in a pilot-scale. The developed technology provides a final virginiamycin titer at the level of 5.6 g/L and has several additional advantages. First, VKM Ac-2738D produces virginiamycin M1 and S1 at the optimum ratio providing the maximum antimicrobial activity of a final product; the performed scale-up kept this important parameter unchanged. Second, VKM Ac-2738D is able to utilize sucrose, which represents less expensive carbon source than traditionally used glucose or d-maltose. Third, the used adsorbing resin, Diaion^®^ HP21, is able to selectively bind up to 98.5% of virginiamycin from culture broth that simplifies the further virginiamycin recovery procedure. The data obtained during this study provide some new information and can be used in other virginiamycin-related studies.
